# Dynamic Analysis of Bi-Stable Galloping Energy Harvesters Under Random Excitation

**DOI:** 10.3390/mi17030358

**Published:** 2026-03-15

**Authors:** Ying Zhang, Ruobing Qin, Kaixin Zheng, Jingwen Zhang

**Affiliations:** 1 School of Mathematics and Statistics, Northwestern Polytechnical University, Xi’an 710072, China; qinruobing@mail.nwpu.edu.cn (R.Q.); 2021204627@mail.nwpu.edu.cn (K.Z.); 2023204882@mail.nwpu.edu.cn (J.Z.); 2 MOE Key Laboratory for Complexity Science in Aerospace, Northwestern Polytechnical University, Xi’an 710072, China

**Keywords:** galloping energy harvester, random excitation, stochastic averaging method, dynamic response

## Abstract

The flow-induced vibration energy harvester provides a solution to the power supply problem for low-power sensors. However, in practical engineering applications, flow-induced vibration energy harvesters often operate in complex environments and are inevitably affected by random external disturbances. Therefore, it is necessary to study the dynamic response of flow-induced vibration energy harvesters under random excitations. In this paper, a bi-stable galloping piezoelectric energy harvesting system is transformed into an equivalent decoupled system through variable transformation. The stochastic averaging method (SAM) of an energy envelope is used to calculate the energy harvester response under random excitation. The validity of the proposed theoretical framework is further confirmed through Monte Carlo (MC) simulations, followed by a systematic analysis of the effects of key parameters on the mean-square voltage output. The results show that noise intensity, aerodynamic coefficient, stiffness coefficient, and wind speed have significant effects on the dynamic response of the system.

## 1. Introduction

In recent years, wireless sensor networks (WSNs) and micro-electro-mechanical systems (MEMS) have advanced rapidly, achieving significant progress in environmental monitoring, intelligent transportation, military applications, and related fields [1,2]. Technologies such as vehicular ad hoc networks (VANETs), wearable electronics, the Internet of Things (IoT), and body area networks (BANs) have been widely applied in precision agriculture and ecological monitoring, smart healthcare and wearable health monitoring, intelligent transportation systems (ITS) with vehicle-road coordination, and aerospace engineering monitoring, with their roles expected to become increasingly critical in the future. However, the exponential increase in wireless sensor nodes and MEMS devices has rendered the long-term energy supply for self-powered microelectronics a persistent challenge in both academia and industry [3,4,5,6,7]. Research on vibration energy harvesting (VEH) aims to address the self-powering issues of such low-power devices. Energy conversion encompasses various mechanisms such as electromagnetic, electrostatic, piezoelectric, and hybrid energy harvesters [8,9,10,11]. Piezoelectric energy harvesters (PEHs) provide advantages such as low cost, stability, reliability, and high output voltage [12]. Researchers in self-powering have extensively investigated and improved PEHs.

PEHs utilize a variety of mechanical vibrations as energy sources, including rotational vibration, harmonic excitation, random excitation, and flow-induced vibration. Flow-induced vibration can be classified into several categories, including vortex-induced vibration [13], galloping [14], wake-induced vibration [15], flutter [16] and mixed flow-induced vibration [17]. Moreover, the theoretical model of galloping was established by A. Barrero-Gil et al. [18], laying the groundwork for potential applications in fluid energy harvesting. Galloping is a classic example of divergent self-excited vibration, resulting from the negative slope of lift and angle of attack curves. This negative damping leads to the absorption of energy from the surroundings, resulting in the generation of divergent vibration [19]. Abdelkefi [20] proposed a PEH with a triangular cross-section and investigated the effects of electrical load impedance on the starting velocity of galloping, lateral displacement, and voltage output level. Yan et al. [21] proposed an innovative hybrid energy harvester for broadband energy conversion. Ref. [22] proposed a galloping PEH with a curved blade turbulence structure, inspired by the trembling of aspen leaves. Liu et al. [23] designed a Y-shaped blunt body composed of three rigid flakes to enhance the efficiency of wind-energy harvesting. Nonlinear factors significantly enhance the efficiency of energy harvesters compared to linear systems. Erturk and Stanton et al. [24,25] investigated the large amplitude periodic oscillation of a bi-stable energy harvester with a magnetic structure. Zhou et al. [26] investigated the dynamic mechanism of an asymmetric tri-stable energy harvester, revealing the critical role of barrier height in determining inter-well vibrations. Yang et al. [27] demonstrated that the nonlinear structure outperforms the linear two-beam structure, resulting in a substantial reduction in galloping initial velocity. Wang et al. [28] developed a new tri-stable galloping PEH, showing that the wind speed threshold is reduced by 33% compared to the traditional model’s threshold. Zhao [29] demonstrated that the introduction of bi-stability effectively expands the operating bandwidth of the system, particularly when wind and foundation vibrations coexist. Li et al. [30] used magnetic coupling to reduce structural stiffness, enabling large-amplitude vibrations at low wind speeds.

However, most existing studies on galloping energy harvesters are based on the assumption of uniform and steady wind flow. In realistic outdoor environments, wind flow is inherently turbulent and characterized by stochastic fluctuations due to complex meteorological conditions and obstacles. These random perturbations can significantly alter the dynamic behavior of nonlinear systems, potentially inducing phenomena such as stochastic resonance or coherence resonance, which deterministic models fail to predict. Therefore, neglecting the stochastic nature of wind excitation may lead to inaccurate estimations of the harvester’s performance in practical applications. To address this issue, researchers have investigated the dynamic response of energy harvesters under random excitations. Daqaq [31] demonstrated the necessity of designing an appropriate potential function to match noise intensity in the presence of nonlinearity in the restoring force. Qin et al. [32] demonstrated that coherence resonance occurs when broadband vibration energy can be transformed into a large-amplitude narrowband low-frequency oscillation response. Xie et al. [33] demonstrated that asymmetric systems outperform traditional symmetric bi-stable systems. Xu et al. [34] conducted a theoretical study on the system response of asymmetric PEHs under the influence of collision factors and random excitation. Sun et al. [35] proposed a novel dual-magnet tri-stable PEH with a single external magnet. The results indicate that, as the excitation level increases, the system transitions from within the trap to a position between the traps.

Considering that realistic wind is a stochastic pulsating excitation rather than deterministic, this study investigates the coupling dynamics of nonlinearity and random excitation. An analytical framework is established to reveal the mechanism of noise-enhanced harvesting. The rest of the paper is arranged as follows. In Section 2, the model of a bi-stable galloping PEH under Gaussian white noise excitation is presented, and the system is transformed into an equivalent single degree of freedom system through harmonic transformation. In Section 3, by means of the stochastic averaging method (SAM), the expression of the stationary probability density of mean square voltage and displacement and velocity is obtained. In Section 4, the effectiveness of the introduced theoretical methods is verified by the relatively good consistency of the numerical and theoretical results. And in order to help to enhance energy harvester performance, the effects of the noise intensity, average wind speed, the aerodynamic coefficient, and the stiffness coefficient on the mean square voltage are explored and analyzed. Finally, concluding remarks are provided in Section 5.

## 2. Model and the Equivalent System

### 2.1. The Bi-Stable Galloping PEH Model

**Figure 1:**
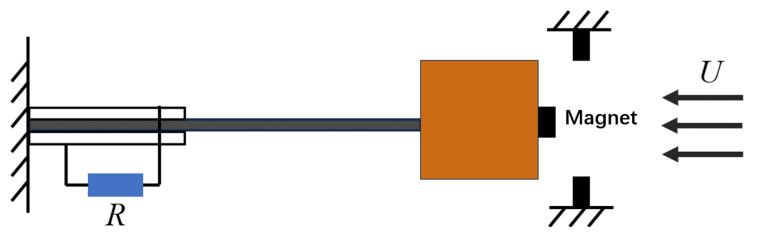
Simplified model of bi-stable galloping piezoelectric energy harvester.

A simplified model of the system is shown in Figure 1. Through theoretical analysis, the electromechanical coupling equation of the bi-stable galloping PEH is derived as [36]:

(1) $$M\ddot{\bar{x}}+C\dot{\bar{x}}+\frac{d\Upsilon }{d\bar{x}}-\bar{\theta }\bar{\nu }=F_{\bar{x}},$$

(2) $$C_{p}\dot{\bar{\nu }}+\frac{\bar{\nu }}{R}+\bar{\theta }\dot{\bar{x}}=0,$$

where *M* is the equivalent mass of the system, $\bar{x}$ is the lateral displacement of the blunt body at the end of the tail, $\bar{\nu }$ is the output voltage of the system, *C* is the equivalent damping of the system, $\bar{\theta }$ is the electromechanical coupling coefficient, $C_{p}$ is the capacitance of the piezoelectric element, and Υ is the potential energy function of the system. In this paper, we consider the most common quadratic form $\Upsilon =\bar{\mu }\;{\bar{x}}^{2}/2+\bar{\gamma }\;{\bar{x}}^{4}/4.$ The potential function contains the intrinsic stiffness of the system and the stiffness change due to the external magnet, so the linear and cubic stiffness term coefficients can be written as $\bar{\mu }={\bar{\mu }}_{i}+{\bar{\mu }}_{e}$ and $\bar{\gamma }={\bar{\gamma }}_{i}+{\bar{\gamma }}_{e}$, where the subscripts *i* and *e* denote the intrinsic and external components, respectively. $F_{\bar{x}}$ represents the galloping aerodynamic force, given by the following expression when considering only linear and cubic nonlinear contributions [37]:

(3) $$F_{\bar{x}}=\frac{1}{2}\rho {\bar{U}}^{2}Ld\left[a_{1}\frac{\dot{\bar{x}}}{\bar{U}}-a_{3}{\left(\frac{\dot{\bar{x}}}{\bar{U}}\right)}^{3}\right],$$

where ρ is the air density and $\bar{U}$ is the wind speed. Respectively, *L* and *d* are the height and width of the blunt body. $a_{1}$ and $a_{3}$ denote aerodynamic force coefficients, which can be experimentally identified through the following wind tunnel tests and are influenced by the cross-sectional geometry and material of the blunt body [38]. Then, we introduce a dimensionless transformation:

$$\omega_{n}=\sqrt{\bar{\mu }/M}\;,\;t=\omega_{n}\tau \;,\;X=\bar{x}/d\;,\;V=C_{p}\bar{\nu }/\left(\bar{\theta }d\right)\;,\;m=\rho Ld^{2}/\left(4M\right)\;,\;\xi_{m}=C/\left(2M\omega_{n}\right)\;,$$

$$U=\bar{U}/\left(\omega_{n}d\right)\;,\;\theta ={\bar{\theta }}^{2}/\left(M\omega_{n}^{2}C_{p}\right)\;,\;\alpha =1/\left(RC_{p}\omega_{n}\right)\;,\;\mu =1+{\bar{\mu }}_{e}/{\bar{\mu }}_{i}\;,\;\gamma =\bar{\gamma }\;d^{2}/{\bar{\mu }}_{i}\;.$$

Considering the inevitable influence of external random excitations in the environmental conditions on energy harvesters, Gaussian white noise ξ(t) with noise intensity *D* is introduced as the stochastic excitation in this study [31]. Admittedly, in realistic wind-driven systems, flow turbulence often exhibits colored (correlated) characteristics and acts multiplicatively through aerodynamic force fluctuations. However, in this study, the stochastic excitation is modeled as an additive Gaussian white noise based on fundamental physical and mathematical considerations. Physically, when the correlation time of the realistic environmental disturbances is significantly shorter than the relaxation time of the harvester, the excitation can be effectively approximated as a Gaussian white noise process [31,39]. Given the typically light mechanical damping of the target galloping harvester, its relaxation time is sufficiently long to satisfy this time-scale separation under assumed weak turbulence scenarios. This additive noise serves as an idealized background disturbance that primarily provides the necessary energy to assist the system in crossing potential barriers. Methodologically, the white-noise assumption is crucial because it ensures that the system response behaves as a Markov process. This allows for the rigorous derivation of the Fokker-Planck-Kolmogorov (FPK) equation and ensures the mathematical tractability required by the energy-envelope stochastic averaging method, intentionally avoiding the intractable parameter-coupling complexity introduced by multiplicative or colored noise. The validity of this additive white-noise approximation is primarily limited to scenarios with weak turbulence and short correlation times. The complex effects of strong turbulence involving colored and multiplicative noise represent an important direction for our future research. Qualitatively, the introduction of colored or multiplicative noise would render the system dynamics non-Markovian, necessitating an augmented state-space representation and a modified Fokker–Planck–Kolmogorov equation to account for the correlation effects and parameter-coupling complexity. Hence, the dimensionless equations for the bi-stable galloping PEH under stochastic excitations are derived as follows:

(4) $$\ddot{X}+2\left(\xi_{m}-ma_{1}U\right)\dot{X}+2\frac{ma_{3}}{U}{\dot{X}}^{3}+\mu X+\gamma X^{3}-\theta V=\xi \left(t\right),$$

(5) $$\dot{V}+\alpha V+\dot{X}=0,$$

where *X* and *V* are dimensionless displacements and voltages, respectively. $\xi_{m}$ is the mechanical damping coefficient. *m* is the fluid and energy harvester inertia ratio. *U* is the dimensionless wind speed. μ and γ are the dimensionless stiffness coefficients, modulated by the position of the external magnets. θ is the dimensionless electromechanical coupling coefficient and α is the time constant ratio. ξ(t) is Gaussian white noise, satisfying $\langle \xi (t)\rangle =0,\langle \xi (t)\xi (t+s)\rangle =2D\delta \left(s\right)$, where *D* is the noise intensity introduced above. Integrating Equations (4) and (5), the analytical expression for voltage V(t) is derived as:

(6) $$V\left(t\right)=C_{1}e^{-\alpha t}+\int_{0}^{t}\dot{X}\left(u\right)e^{-\alpha (t-u)}du,$$

where the integration constant $C_{1}$ depends on the initial conditions, the decay term $C_{1}e^{-\alpha t}$ can be neglected in the steady state case by considering the smooth output voltage of the system. Considering the steady-state case, the voltage expression in Equation (6) can be simplified to Equation (7):

(7) $$V\left(t\right)=\int_{0}^{t}\dot{X}\left(u\right)e^{-\alpha (t-u)}du.$$

### 2.2. Equivalent System

Let the solutions of Equations (4) and (5) be:

(8) $$X\left(t\right)=A\left(t\right)\cos\Phi ,\dot{X}\left(t\right)=-A\left(t\right)\omega_{0}\sin\Phi ,$$

where $\Phi \left(t\right)=\omega_{0}t+\varphi \left(t\right)$, A(t) is the amplitude, Φ(t) is the phase angle. Substituting Equation (8) into Equation (7) yields Equation (9):

(9) $$V\left(t\right)=-\frac{\omega_{0}A\left(t\right)}{\alpha^{2}+\omega_{0}^{2}}\left(\omega_{0}\cos\Phi -\alpha \sin\Phi \right)=-\frac{\omega_{0}^{2}}{\alpha^{2}+\omega_{0}^{2}}X\left(t\right)-\frac{\alpha }{\alpha^{2}+\omega_{0}^{2}}\dot{X}\left(t\right).$$

The amplitude of the steady state voltage can be expressed as $V_{\max}=\omega_{0}A\left(t\right)/\sqrt{\alpha^{2}+{\omega_{0}}^{2}}$. Based on Equation (9), the equivalent decoupled system of Equations (1) and (2) is expressed as:

(10) $$\ddot{X}+\left(2\xi_{m}-2ma_{1}U+\frac{\theta \alpha }{\alpha^{2}+{\omega_{0}}^{2}}\right)\dot{X}+2\frac{ma_{3}}{U}{\dot{X}}^{3}+\left(\mu +\frac{\theta {\omega_{0}}^{2}}{\alpha^{2}+{\omega_{0}}^{2}}\right)X+\gamma X^{3}=\xi \left(t\right),$$

where, $\omega_{0}$ represents the expected fundamental oscillation frequency of the system under the narrow-band random response. Through the harmonic ansatz, the electromechanical coupling equivalently introduces an electrical damping term $\theta \alpha /(\alpha^{2}+\omega_{0}^{2})$ and an electrical stiffness term $\theta \omega_{0}^{2}/\left(\alpha^{2}+\omega_{0}^{2}\right)$ into the mechanical equation. It should be noted that this harmonic ansatz (X(t)=A(t)cosΦ) used for the decoupling is strictly valid under the assumptions of light mechanical damping and weak electromechanical coupling. Under these conditions, the overall energy envelope evolves much more slowly than the rapid oscillatory phase, allowing the narrow-band approximation to hold for predicting stationary statistics, even if the inter-well snap-through motion is non-sinusoidal. It should be explicitly noted that, when generating the analytical results across various parameter sweeps, $\omega_{0}$ is practically pre-set to a fixed constant (specifically, $\omega_{0}=2$ in the present dimensionless analysis). The mathematical justification for this constant approximation lies in the weak sensitivity of the decoupled system. As shown in Equation (10), $\omega_{0}$ exclusively appears within the equivalent electrical damping and electrical stiffness terms. Because the galloping energy harvester operates in a weakly coupled regime (e.g., θ≈0.13), these electrical terms act as minor perturbations compared to the dominant mechanical damping and stiffness. Consequently, even if the actual oscillation frequency fluctuates dynamically under varying environmental conditions, the resulting mathematical variation in these already minor electrical terms is a negligible higher-order small quantity. This theoretically weak sensitivity justifies that treating $\omega_{0}$ as a fixed constant is a highly accurate first-order approximation, avoiding the intractable complexity of case-by-case numerical frequency extraction without compromising the fidelity of the theoretical predictions.

## 3. The Stochastic Averaging Method

From Equation (10), the decoupled mechanical equation can be rewritten in a compact form:

(11) $$\ddot{X}+c_{1}\dot{X}+c_{3}{\dot{X}}^{3}+\delta_{1}X+\delta_{2}X^{3}=\xi \left(t\right),$$

where the equivalent stiffness coefficients ($\delta_{1},\delta_{2}$) and equivalent damping coefficients ($c_{1},c_{3}$) are given by Equation (12):

(12) $$\begin{array}{cccc} \delta_{1} & =\mu +\frac{\theta \omega_{0}^{2}}{\alpha^{2}+\omega_{0}^{2}},\; & \delta_{2} & =\gamma ,\\ c_{1} & =2\xi_{m}-2ma_{1}U+\frac{\theta \alpha }{\alpha^{2}+\omega_{0}^{2}},\; & c_{3} & =\frac{2ma_{3}}{U}. \end{array}$$

The potential function Λ(X) and total energy $H(X,\dot{X})$ of the system are defined as:

(13) $$\Lambda \left(X\right)=\frac{\delta_{1}}{2}X^{2}+\frac{\delta_{2}}{4}X^{4}+\frac{\delta_{1}^{2}}{4\delta_{2}},$$

(14) $$H\left(X,\dot{X}\right)=\frac{{\dot{X}}^{2}}{2}+\Lambda \left(X\right).$$

The period of the system T(H) over one complete cycle depends on the energy level *H*. Specifically, it is evaluated in two distinct regimes:

For the intra-well motion ($H\leq \frac{\delta_{1}^{2}}{4\delta_{2}}$), the expected period is given by:

(15) $$T\left(H\right)=2\int_{A_{H1}}^{A_{H2}}\frac{dX}{\sqrt{2H-2\Lambda (X)}}.$$

For the inter-well snap-through motion ($H>\frac{\delta_{1}^{2}}{4\delta_{2}}$), the expected period becomes:

(16) $$T\left(H\right)=4\int_{0}^{A_{H2}}\frac{dX}{\sqrt{2H-2\Lambda (X)}}.$$

where $A_{H1}$ and $A_{H2}$ represent the minimum and maximum amplitudes, respectively, obtained when H=Λ(X). Specifically, depending on the energy level *H*, these turning points are explicitly given in the following forms.

For $H\leq \frac{\delta_{1}^{2}}{4\delta_{2}}$, both turning points exist:

(17) $$A_{H1}=\sqrt{\frac{-\delta_{1}-\sqrt{\delta_{1}^{2}+4\delta_{2}\left(H+\frac{\delta_{1}^{2}}{4\delta_{2}}\right)}}{\delta_{2}}},\;A_{H2}=\sqrt{\frac{-\delta_{1}+\sqrt{\delta_{1}^{2}+4\delta_{2}\left(H+\frac{\delta_{1}^{2}}{4\delta_{2}}\right)}}{\delta_{2}}}.$$

For $H>\frac{\delta_{1}^{2}}{4\delta_{2}}$, only one positive turning point exists:

(18) $$A_{H2}=\sqrt{\frac{-\delta_{1}+\sqrt{\delta_{1}^{2}+4\delta_{2}\left(H+\frac{\delta_{1}^{2}}{4\delta_{2}}\right)}}{\delta_{2}}}.$$

The integration limits and coefficients strictly depend on the motion regimes of the bi-stable system. For the intra-well motion ($H\leq \delta_{1}^{2}/\left(4\delta_{2}\right)$), the oscillator is confined within one of the potential wells, and the integration is performed between the inner root $A_{H1}$ and outer root $A_{H2}$ with a coefficient of 2 representing a full cycle. For the inter-well motion ($H>\delta_{1}^{2}/\left(4\delta_{2}\right)$), the oscillator crosses the potential barrier, and the integration is performed from the center 0 to the maximum amplitude $A_{H2}$ with a coefficient of 4 due to the symmetry of the phase space trajectory.

Based on the energy definition, the derivatives of the state variables with respect to time yield:

(19) $$\begin{array}{c} \dot{X}=\pm \sqrt{2H-2\Lambda (X)},\\ \dot{H}=-\left(c_{1}{\dot{X}}^{2}+c_{3}{\dot{X}}^{4}\right)+\dot{X}\xi \left(t\right). \end{array}$$

When the damping and noise intensity are small, according to the energy-envelope stochastic averaging method, the state response can be approximated as a one-dimensional Markov diffusion process. The Itô stochastic differential equation for the energy *H* is given by:

(20) dH=m(H)dt+σ(H)dB(t),

where B(t) is a standard Wiener process. m(H) and σ(H) are the drift and diffusion coefficients, respectively, which are derived as:

(21) $$\begin{array}{c} m\left(H\right)={\langle -c_{1}{\dot{X}}^{2}-c_{3}{\dot{X}}^{4}\rangle }_{t}+D,\\ \sigma^{2}\left(H\right)=2D{\langle {\dot{X}}^{2}\rangle }_{t}. \end{array}$$

where, the time-averaging operator ${\langle \left[\cdot \right]\rangle }_{t}$ over one oscillation cycle is rigorously defined based on the energy level *H*:

(22) $${\langle \left[\cdot \right]\rangle }_{t}=\left\{\begin{array}{cc} \frac{2}{T(H)}\int_{A_{H1}}^{A_{H2}}\frac{{\left[\cdot \right]}_{\dot{X}=\sqrt{2H-2\Lambda (X)}}}{\sqrt{2H-2\Lambda (X)}}dX, & \;H\leq \frac{\delta_{1}^{2}}{4\delta_{2}},\\ \frac{4}{T(H)}\int_{0}^{A_{H2}}\frac{{\left[\cdot \right]}_{\dot{X}=\sqrt{2H-2\Lambda (X)}}}{\sqrt{2H-2\Lambda (X)}}dX, & \;H>\frac{\delta_{1}^{2}}{4\delta_{2}}. \end{array}\right.$$

Therefore, the Fokker-Planck-Kolmogorov (FPK) equation associated with Equation (20) is expressed as:

(23) $$\frac{\partial p}{\partial t}=-\frac{\partial }{\partial H}\left(m\left(H\right)p\right)+\frac{1}{2}\frac{\partial^{2}}{\partial H}\left(\sigma^{2}\left(H\right)p\right).$$

Considering the steady state solution response of the system, the solution of Equation (23) can be expressed as [40]:

(24) $$p\left(H\right)=\frac{C}{\sigma^{2}\left(H\right)}\exp\left\{2\int \frac{m(H)}{\sigma^{2}\left(H\right)}dH\right\}.$$

where *C* is a normalizing constant. Considering the relationship among *X*, $\dot{X}$ and *H*, the joint and marginal probability density functions (PDFs) of displacement and velocity are obtained:

(25) $$p\left(X,\dot{X}\right)=\frac{p(H)}{T(H)}{|}_{H=\frac{1}{2}{\dot{X}}^{2}+\Lambda \left(X\right)},\;p\left(X\right)=\int_{-\infty }^{+\infty }p\left(X,\dot{X}\right)d\dot{X},\;p\left(\dot{X}\right)=\int_{-\infty }^{+\infty }p\left(X,\dot{X}\right)dX.$$

According to Equations (9) and (25), the steady state mean square voltage is given by:

(26) $$E\left[V^{2}\right]={\left(\frac{\omega_{0}^{2}}{\alpha^{2}+\omega_{0}^{2}}\right)}^{2}E\left[X^{2}\right]+{\left(\frac{\alpha }{\alpha^{2}+\omega_{0}^{2}}\right)}^{2}E\left[{\dot{X}}^{2}\right]+2\frac{\omega_{0}^{2}\alpha }{{\left(\alpha^{2}+\omega_{0}^{2}\right)}^{2}}E\left[X\dot{X}\right].$$

where

(27) $$E\left[X^{2}\right]=\int_{-\infty }^{+\infty }X^{2}p\left(X\right)dX,\;E\left[{\dot{X}}^{2}\right]=\int_{-\infty }^{+\infty }{\dot{X}}^{2}p\left(\dot{X}\right)d\dot{X},\;E\left[X\dot{X}\right]=\int_{-\infty }^{+\infty }\int_{-\infty }^{+\infty }X\dot{X}p\left(X,\dot{X}\right)dXd\dot{X}.$$

In the context of piezoelectric energy harvesting, the average harvested power is a more direct and critical performance metric than the output voltage alone. In the physical domain, the average harvested power across the load resistance *R* is defined as $P_{real}=E\left[{\bar{\nu }}^{2}\right]/R$. Recalling the dimensionless transformations defined in Section 2.1, the physical voltage and load resistance can be expressed in terms of their dimensionless counterparts as $\bar{\nu }=\left(\bar{\theta }d/C_{p}\right)V$ and $1/R=\alpha C_{p}\omega_{n}$. Substituting these relations into the physical power expression yields:

(28) $$P_{real}=\frac{1}{R}E\left[{\bar{\nu }}^{2}\right]=\left(\alpha C_{p}\omega_{n}\right){\left(\frac{\bar{\theta }d}{C_{p}}\right)}^{2}E\left[V^{2}\right]=\left[\frac{{\left(\bar{\theta }d\right)}^{2}\omega_{n}}{C_{p}}\right]\alpha E\left[V^{2}\right].$$

The term in the square brackets comprises solely the intrinsic physical constants of the system and carries the unit of Watt. Therefore, by isolating the dimensionless variables, the dimensionless average harvested power $P_{\mathrm{avg}}$ can be explicitly and naturally defined as:

(29) $$P_{\mathrm{avg}}=\alpha E\left[V^{2}\right].$$

Since α is the reciprocal of the dimensionless electrical time constant, investigating the variation of $P_{\mathrm{avg}}$ with respect to α reflects the load dependence of the energy harvester.

## 4. Results and Discussion

In this section, the influence of system parameters on the stochastic response is discussed. Equations (4) and (5) are solved using a numerical method and compared with the theoretical results to verify the validity of the analytical method and to quantify the approximation error introduced by the electromechanical decoupling. To ensure the full reproducibility of the numerical results, the fully coupled electromechanical equations (Equations (4) and (5)) are numerically integrated using an explicit stochastic Runge-Kutta (SRK) algorithm in the Itô sense. Because the system is driven by additive white noise, this explicit scheme rigorously satisfies the strong order assumptions to provide an accurate approximation for the state trajectories [41]. The dimensionless integration time step is strictly set to Δt=0.011 to guarantee the convergence of the numerical scheme. The total dimensionless simulation time is *T* = 99,000, yielding approximately $9\times 10^{6}$ integration steps for one long realization. To thoroughly eliminate the influence of initial transient behaviors and ensure that the system has reached a stationary state, the data from the initial phase are discarded, and only the steady-state time series are retained for statistical analysis. Furthermore, the stationary probability density functions (PDFs) are estimated using the histogram method with 100 bins. The numerical convergence has been explicitly verified through a two-fold check: first, halving the integration time step from Δt=0.011 to 0.0055 (while doubling the total number of steps to maintain the same simulation time *T*) results in no significant change in the mean-square voltage $E[V^{2}]$ (with a relative difference of less than 0.01%); second, refining the histogram from 100 to 200 bins yields visually indistinguishable PDF curves. These results confirm that the chosen time step and statistical resolution are sufficient to provide stable and converged stationary results. The initial conditions are set as X(0)=0, $\dot{X}\left(0\right)=0$ and V(0)=0. The system parameters are chosen as follows: $\xi_{m}=0.003$, m=0.006, $a_{1}=2.5$, $a_{3}=130$, θ=0.13, α=5.5, D=0.01.

### 4.1. Stochastic Response Characteristics

**Figure 2:**
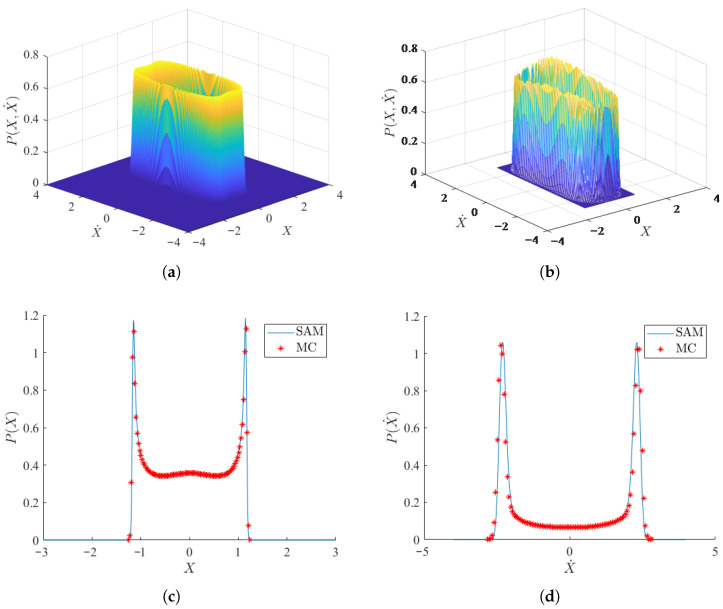
Stochastic responses of the system with initial conditions $X\left(0\right)=0,\dot{X}\left(0\right)=0,V\left(0\right)=0$ and parameters $\xi_{m}=0.003,m=0.006,a_{1}=2.5,a_{3}=130,\theta =0.13,\alpha =5.5,D=0.01$. (**a**) Theoretical results of the joint density function of the system displacement and velocity; (**b**) Numerical results of the joint density function of the system displacement and velocity; (**c**) Steady-state probability density function of the displacement *X*; (**d**) Steady-state probability density function of the velocity $\dot{X}$. In (**c**,**d**), the solid lines denote analytical results obtained by SAM and the scattered markers denote numerical results obtained by MC simulations.

A comparison between the theoretical and numerical solutions of the probability density function (PDF) of the system is shown in Figure 2, demonstrating the good agreement between the results of the two methods. This validates the efficacy of the energy envelope stochastic averaging method for this type of problem. Furthermore, the displacement and velocity of the system clearly exhibit bi-stable characteristics.

**Figure 3:**
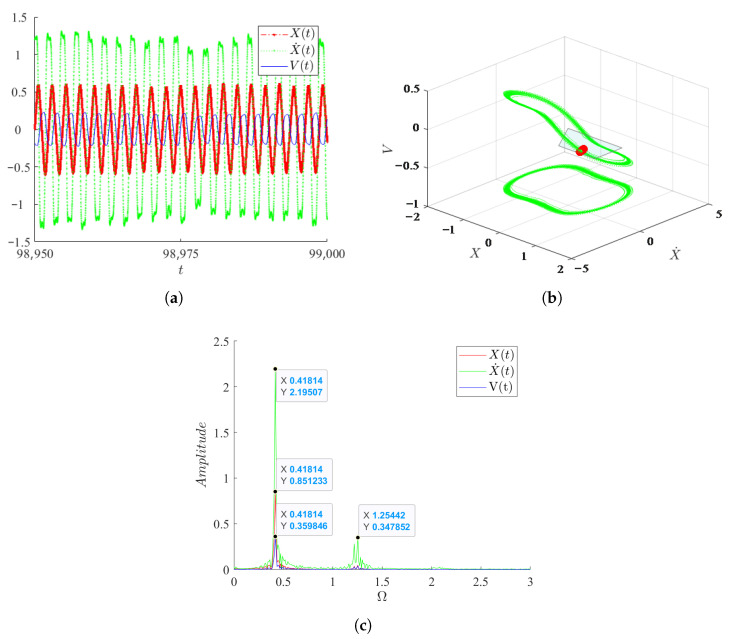
Dynamic responses of the three system variables: displacement *X*, velocity $\dot{X}$, and voltage *V*. (**a**) The time history responses of the displacement *X*, velocity $\dot{X}$, and voltage *V*; (**b**) Phase diagram showing a limit cycle motion; (**c**) The spectrogram of *X*, $\dot{X}$ and *V* highlighting the dominant vibrational frequencies.

The response of the three variables of the system is shown in Figure 3. It is observed that, in comparison to velocity, the displacement and output voltage exhibit greater stability, although the response amplitudes of all three variables exhibit fluctuations. Notably, short-term fluctuations are observed near the amplitude of the system’s velocity. The spectrogram reveals that the response frequencies of the system’s three variables are predominantly concentrated around 0.41, with varying peak values. The response frequency of velocity exhibits two peaks near 1.25 in addition to 0.41, corresponding to the fluctuation phenomenon observed at each peak in the time series plot of velocity. The phase diagram of the system forms a limit cycle, with the Poincaré cross-section points forming a tightly packed string. When projected onto a two-dimensional plane, it becomes evident that the system exhibits periodic motion between two steady states.

**Figure 4:**
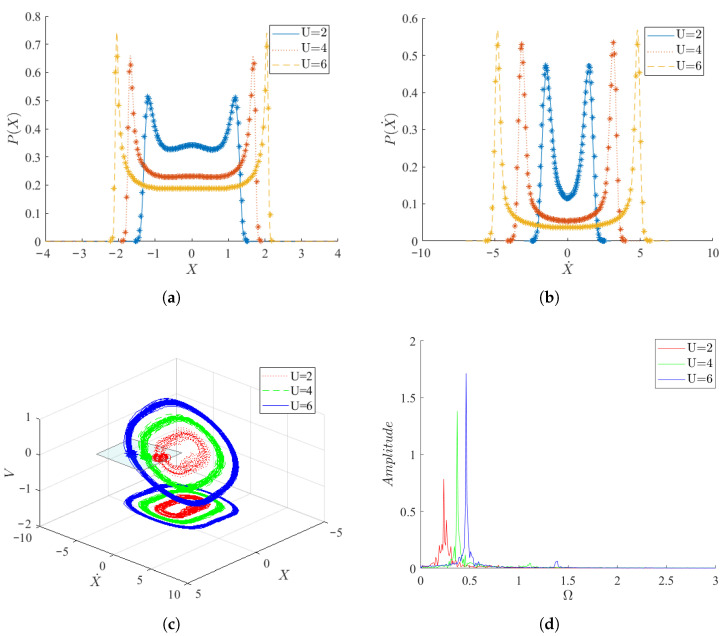
Response of the system for different wind speeds *U* (μ=−1,γ=3,D=0.05). (**a**) Steady state density function of displacement *X*; (**b**) Steady state density function of velocity $\dot{X}$; (**c**) Phase diagram under different wind speeds; (**d**) The spectrogram of displacement *X*. In the steady state density function (**a**,**b**), the solid lines denote analytical results obtained by SAM and the scattered markers denote numerical results obtained by MC simulations.

**Figure 5:**
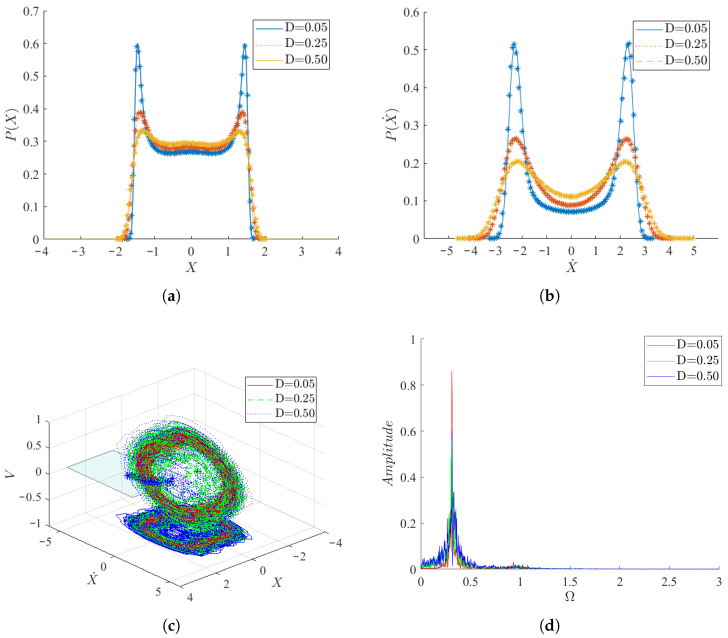
Responses of the system for different noise intensities *D* (μ=−1,γ=3,U=3). (**a**) Steady-state probability density function of displacement *X*; (**b**) Steady-state probability density function of velocity $\dot{X}$; (**c**) Phase diagram showing the transition to chaotic motion; (**d**) The spectrogram of displacement *X*. In the steady state density function (**a**,**b**), the solid lines denote analytical results obtained by SAM and the scattered markers denote numerical results obtained by MC simulations.

As indicated in Figure 4 and Figure 5, the vibration of the system is governed by the aerodynamic force $F_{x}$ and the random excitation ξ(t). Therefore, the effects of mean wind speed, aerodynamic coefficient, stiffness coefficient, and random excitation intensity on the PDF and mean square voltage will be investigated in the following. When μ=−1,γ=3,D=0.05, Figure 4 illustrates the changes in the PDFs of system displacement and velocity as the wind speed gradually increases. With increasing wind speed, the system transitions from small amplitude motion to a trend of large amplitude motion. Simultaneously, the vibration velocity of the system significantly increases, along with a gradual increase in the peak values of the two density functions. The phase diagrams reveal that, at all three wind speeds, the system forms limit cycles. Additionally, the limit cycles corresponding to higher wind speeds encompass those corresponding to lower wind speeds in all three dimensions of displacement, velocity, and voltage. Spectrograms indicate that the dominant vibrational frequency of the system displacement, along with its corresponding peak value, increases with increasing wind speed. Consequently, larger wind speeds not only increase the vibration frequency and amplitude of the system but also render the vibration more regular, thereby enhancing the performance of the PEH.

When μ=−1,γ=3,U=3, Figure 5 shows the changes in the PDFs of system displacement and velocity as the noise intensity. As the noise intensity increases, the peak values of the PDF of the system displacement and velocity gradually decrease, while the maximum amplitude and velocity will slightly increase. This suggests a gradual increase in displacement and velocity. The phase diagram reveals a gradual transition of the system response from a limit cycle to a chaotic state, with chaotic motion surrounding the limit cycle. The spectrum diagram shows that, as the noise intensity increases, the main frequency of the system displacement remains unchanged, but its corresponding peak value gradually decreases. Meanwhile, the peak value of other frequencies near the main frequency gradually increases. Consequently, the increase in noise intensity leads to a slight increase in the displacement, velocity, and output voltage of the system, accompanied by irregular vibration, resulting in a chaotic system response.

### 4.2. Effects on the Mean Square Voltage

**Figure 6:**
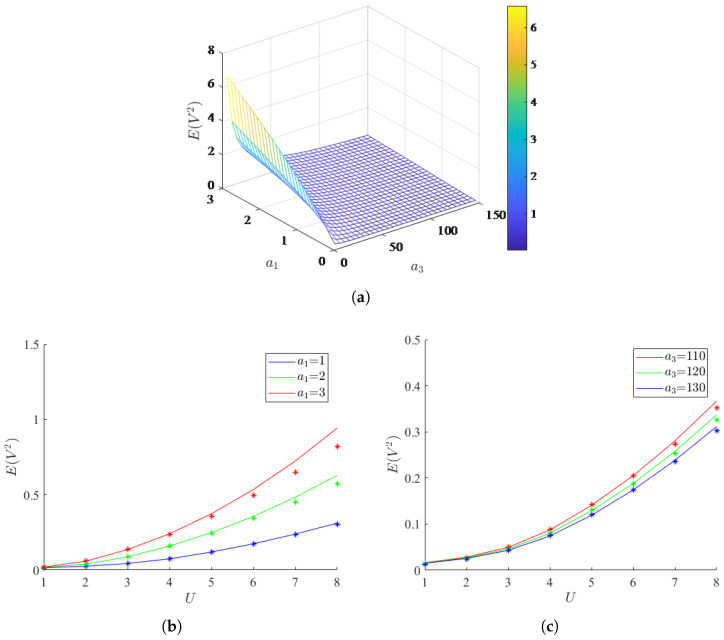
Effects of the aerodynamic coefficients on the mean square voltage (U=5,D=0.1,μ=−1, and γ=1). (**a**) Effect of combined aerodynamic coefficients $a_{1}$ and $a_{3}$; (**b**) Effect of aerodynamic coefficient $a_{1}$; (**c**) Effect of aerodynamic coefficient $a_{3}$. The solid lines denote analytical results obtained by SAM and the scattered markers denote numerical results obtained by MC simulations.

**Figure 7:**
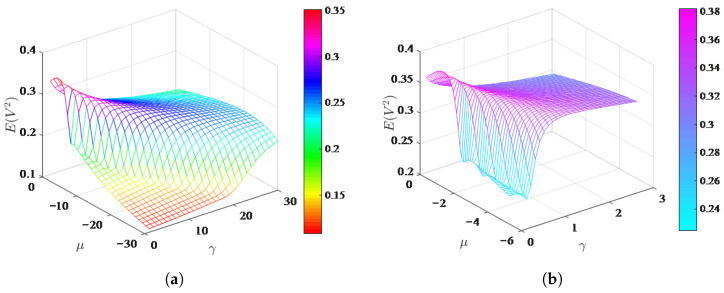
Effects of the stiffness coefficients on the mean square voltage. (**a**) Effect of the joint stiffness coefficients μ and γ on the mean square voltage; (**b**) Localized enlargement of (**a**).

The steady state mean square voltage is a key indicator of the system, and the effect of system parameters on it will be explored in the following. The results obtained from both analytical and numerical methods are compared to demonstrate the accuracy and effectiveness of the analytical method. To better understand the effect of the aerodynamic coefficient on the system, the changes in mean square voltage with respect to the aerodynamic coefficients $a_{1}$ and $a_{3}$ are shown in Figure 6, for U=5,D=0.1,μ=−1, and γ=1. It is evident that larger $a_{1}$ and smaller $a_{3}$ favorably enhance the mean-square voltage. Figure 6b,c presents the variation of the system mean square voltage with wind speed after selecting specific values for $a_{1}$ and $a_{3}$. The increasing spacing between the curves as the wind speed increases suggests that the selection of $a_{1}$ and $a_{3}$ has a growing impact on the performance of the energy harvester. Figure 7 illustrates the variations in mean square voltage under different stiffness factors, where Figure 7b provides a localized enlargement of Figure 7a. As shown in Figure 7, when γ is fixed, the mean square voltage first increases and then decreases as μ gradually decreases. Additionally, the mean square voltage reaches its maximum when μ=−1.2,γ=0.1. However, there is a rapid drop in the mean square voltage when γ=0.1,μ∈[−5,−2]. So, there always exists a suitable μ and γ making the mean square voltage reach its maximum.

**Figure 8:**
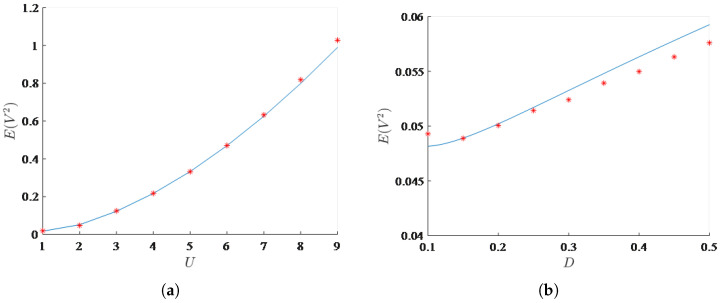
Effects of wind speed and noise intensity on the mean square voltage. (**a**) Effect of wind speed *U* on the mean square voltage; (**b**) Effect of noise intensity *D* on the mean square voltage. The solid lines denote analytical results obtained by SAM, and the scattered markers denote numerical results obtained by MC simulations.

Figure 8a,b shows the variation of mean square voltage with wind speed and noise intensity, respectively. It is evident that both wind speed and noise intensity have a positive impact on the mean square voltage. However, comparatively, wind speed demonstrates a more pronounced enhancement in the mean square voltage compared to noise intensity. Therefore, to significantly enhance the output performance of the energy harvester, consideration should be given to the prevailing ambient wind speed conditions.

Although the above analysis indicates that increasing noise intensity generally enhances the steady-state mean-square voltage by facilitating frequent inter-well snap-through motions, it is crucial to emphasize the inherent trade-offs in practical implementations. In realistic bi-stable energy harvesters, the overall performance dependence on noise intensity can exhibit non-monotonic characteristics. On the one hand, moderate noise optimally assists the galloping oscillator in overcoming the potential barrier, thereby maximizing the harvested energy through stochastic resonance-like behavior. On the other hand, excessive noise intensity exacerbates the irregularity and disorder of the response, broadening the frequency spectrum, which significantly degrades the conversion efficiency of standard alternating current-to-direct current (AC-DC) rectification circuits. Furthermore, from a structural reliability perspective, higher noise levels inevitably induce extreme peak displacements and concentrated mechanical stresses. This drastically increases the risk of structural fatigue and compromises the long-term robustness of the harvester. Therefore, an optimal noise intensity effectively exists in practical scenarios, representing a critical trade-off between promoting energetic barrier-crossing events and maintaining necessary response stability.

**Figure 9:**
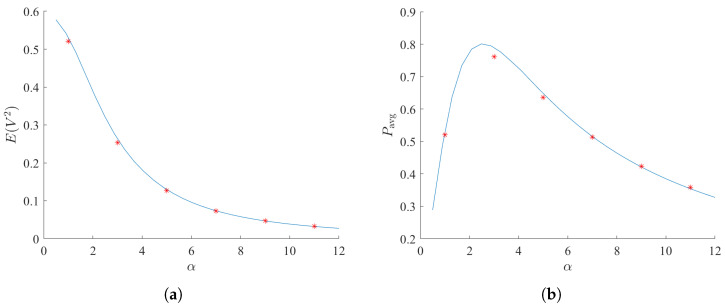
Effects of the electrical time constant ratio on the energy harvesting performance. (**a**) Effect of the electrical time constant ratio α on the mean square voltage $E[V^{2}]$; (**b**) Effect of α on the average harvested power $P_{\mathrm{avg}}$. The solid lines denote analytical results obtained by SAM, and the scattered markers denote numerical results obtained by MC simulations. The fixed system parameters are chosen as U=3,D=0.01,μ=−3, and γ=10.

Finally, to address the practical implementation of the bi-stable galloping energy harvester, the load dependence of the system is thoroughly investigated. Since the electrical time constant ratio α is inversely proportional to the load resistance *R*, the dimensionless average harvested power $P_{\mathrm{avg}}$ depends significantly on this parameter, as indicated by Equation (29). To better understand the effect of the electrical load on the system performance, the variations of the mean square voltage and the harvested power $P_{\mathrm{avg}}$ with respect to α are illustrated in Figure 9, for the fixed parameters U=3,D=0.01,μ=−3, and γ=10.

As α increases (which physically corresponds to a decrease in the load resistance *R*), the mean square voltage exhibits a monotonic decrease, owing to the heavier electrical damping effect. However, the average harvested power $P_{\mathrm{avg}}$ first increases and then decreases, forming a distinct unimodal curve. This phenomenon explicitly indicates that an optimal electrical load exists under stochastic excitation. This optimal value of α achieves the best impedance matching, effectively balancing the energy extracted by the electrical circuit and the equivalent electrical damping introduced back to the mechanical galloping oscillator, thereby maximizing the overall energy conversion efficiency.

## 5. Conclusions

This paper investigates the dynamic behavior of a bi-stable galloping piezoelectric energy harvester under external random excitation. The steady-state PDFs of displacement and velocity, along with the mean square voltage, are derived using the energy enveloping SAM. The effectiveness of the analytical approach is validated by comparing the analytical results with numerical methods.

Based on the parametric analysis, several key conclusions and design guidelines are drawn to enhance the engineering applicability of such devices. The study reveals that increasing wind speed and noise intensity generally lead to a higher mean square voltage. Specifically, external random disturbances play a constructive role in energy harvesting, as an appropriate level of noise intensity can assist the system in crossing the potential barrier, facilitating large-amplitude inter-well oscillations. This suggests that bi-stable harvesters are particularly suitable for turbulent wind environments where flow fluctuations are prevalent. However, while noise contributes to enhancement, the mean wind speed remains the dominant factor. Regarding the aerodynamic profile design, results indicate that a larger unstable aerodynamic coefficient ($a_{1}$) and a smaller aerodynamic damping coefficient ($a_{3}$) significantly improve the output. Therefore, in practical design, bluff body cross-sections exhibiting steeper lift coefficient slopes should be prioritized to maximize aerodynamic instability. Furthermore, optimal adjustment of stiffness characteristics is essential. While bi-stability broadens the operating bandwidth, an excessively deep potential well may trap the system in low-energy intra-well oscillations. Consequently, designers should tune the magnetic distance to create a “shallow” bi-stable potential, ensuring that the available wind energy and random perturbations are sufficient to trigger high-energy snap-through motions. These insights provide a theoretical basis for the customized design of galloping energy harvesters tailored to specific wind environments, balancing the trade-off between structural stability and energy capture efficiency.

## Data Availability

The original contributions presented in this study are included in the article. Further inquiries can be directed to the corresponding author.
